# Semen microbiota are dramatically altered in men with abnormal sperm parameters

**DOI:** 10.1038/s41598-024-51686-4

**Published:** 2024-01-11

**Authors:** Vadim Osadchiy, Andre Belarmino, Reza Kianian, John T. Sigalos, Jacob S. Ancira, Trisha Kanie, Sarah F. Mangum, Craig D. Tipton, Tung-Chin M. Hsieh, Jesse N. Mills, Sriram V. Eleswarapu

**Affiliations:** 1 Division of Andrology, Department of Urology, David Geffen School of Medicine, University of California, 10945 Le Conte Avenue, Ueberroth #3361, Los Angeles, CA 90095 USA; 2 RTL Genomics, MicroGen DX, Lubbock, TX USA; 3 Department of Biological Sciences, Texas Tech University, Lubbock, TX USA; 4 Department of Urology, University of California, San Diego, CA USA

**Keywords:** Microbiology, Urology, Gonads

## Abstract

There has recently been an explosion of studies implicating the human microbiome in playing a critical role in many disease and wellness states. The etiology of abnormal semen analysis (SA) parameters is not identified in 30% of cases; investigations involving the semen microbiome may bridge this gap. Here, we explore the relationship between the semen microbiome and alterations of sperm parameters. We recruited men presenting for fertility evaluation or vasectomy consultation with proven biological paternity. SA and next generation sequencing was performed. Differential abundance testing using Analysis of composition of Microbiota with Bias Correction (ANCOM-BC) was performed along with canonical correlational analysis for microbial community profiling. Men with abnormal (N = 27) sperm motility showed a higher abundance of *Lactobacillus iners* compared to those with normal (N = 46) sperm motility (mean proportion 9.4% versus 2.6%, *p* = 0.046). This relationship persisted on canonical correlational analysis (r = 0.392, *p* = 0.011). Men with abnormal sperm concentration (N = 20) showed a higher abundance of *Pseudomonas stutzeri* (2.1% versus 1.0%, *p* = 0.024) and *Pseudomonas fluorescens* (0.9% versus 0.7%, *p* = 0.010), but a lower abundance of *Pseudomonas putida* (0.5% versus 0.8%, *p* = 0.020), compared to those with normal sperm concentration (N = 53). Major limitations are related to study design (cross-sectional, observational). Our results suggest that a small group of microorganisms may play a critical role in observed perturbations of SA parameters. Some of these microbes, most notably *Lactobacillus iners*, have been described extensively within other, fertility-related, contexts, whereas for others, this is the first report where they have potentially been implicated. Advances in our understanding of the semen microbiome may contribute to potentially new therapeutic avenues for correcting impairments in sperm parameters and improving male fertility.

## Introduction

Male factor infertility is common and, despite advances in semen and genetic testing, still not fully understood^1^. The etiology of abnormal semen analysis (SA) parameters is not identified in 30% of cases^2^. Recent studies have implicated a role for the microbiome in health and human disease^3,4^. These investigations have recently expanded to an exploration of the semen microbiome and its potential role in male factor infertility.

Advances in next-generation sequencing (NGS) technologies have augmented our understanding of human health and disease by allowing for accurate, efficient, and relatively inexpensive characterization of the human microbiome. There have been a small handful of studies that have explored the semen microbiome, and an even smaller handful to explore the semen microbiome within the context of fertility. These studies have largely been limited by their superficial, retrospective nature and small sample sizes^3^. More recently, higher quality studies though still with relatively limited sample sizes have emerged. Lundy et al.^5^ explored the taxonomic and functional profile of the semen microbiome in 32 well-phenotyped men. Their findings underscore differences in alpha and beta diversity among infertile men compared with healthy controls, in addition to a direct association between genus *Pseudomonas* and total motile sperm count. Another study with a similar sample size found that men with non-obstructive azoospermia compared to healthy controls, demonstrate changes in semen microbiome beta diversity and associated taxonomic differences^6^.

Here, we expand on our understanding of the semen microbiome and its role in male infertility and alterations in SA parameters. Although results from a cross-sectional study do not allow us to make inferences related to causality, we hypothesize that a small, but critical group of microorganisms may play an important role in contributing to subfertility in men. Our findings will serve as the foundation for future, mechanistic, and potentially longitudinal studies. This is the largest study to explore this line of inquiry.

## Methods

### Study population and biospecimen collection

Men aged ≥ 18 years who presented either for an initial fertility evaluation (both primary and secondary infertility were included) or men with proven biological paternity prior to their vasectomy consultation were recruited for this study. The recruitment period was from 08/2021 through 06/2022, with an initial aim to recruit approximately 100 participants. All participants provided written informed consent and denied any acute illness. Information regarding participant clinical information was also collected, including age, body mass index, foreskin status, tobacco usage, and alcohol use. Social alcohol use was defined by ≤ 14 drinks per week; heavy alcohol use was > 14 drinks per week. Due to a lack of detailed information related to paternity timeline, some individuals who presented for vasectomy consultation may have fathered children many years ago so their current fertility cannot be guaranteed. Given this, we ultimately stratified our cohorts of interest by SA parameters, reflecting more objective, contemporary data. Semen samples were collected following 2–7 days of abstinence. All specimens were obtained prior to any surgical (including vasectomy) or pharmacologic intervention for fertility. Specimens for microbiome analysis were stored at − 80 °C until they were processed.

### Semen analysis

Semen was evaluated according to World Health Organization 5^th^ Edition criteria using a calibrated SQA-Vision Automated Semen Analyzer (Medical Electronic Systems, Encino, CA). Samples demonstrating oligozoospermia or azoospermia were independently evaluated using high-powered microscopy. Semen volume, pH, concentration, motility, and strict morphology (Kruger) are reported. Total motile sperm count was calculated by multiplying total concentration, total motility, and volume.

### Microbiome community profiling

DNA extraction, PCR amplification, library preparation, and sequencing of 16S rRNA regions V1-V2 on Illumina Miseq platform was conducted as previously performed by MicroGenDX (Lubbock, TX)^7,8^. Briefly, DNA extraction was performed with a Qiagen TissueLyser and Zymo MagBead 96 DNA/RNA kit (Zymo Research, Tustin, CA, USA). Samples were mechanically lysed using Zirconium oxide beads (0.5 mm) and the Qiagen TissueLyser. The lysate was extracted for total DNA following the Zymo MagBead 96 DNA/RNA kit’s protocol on the KingFisher FLEX (ThermoFisher, Grand Island, NY, USA). The samples were amplified for sequencing using a 25uL reaction containing the 28F primer (GAGTTTGATCNTGGCTCAG) with the Illumina adapter and a unique barcode and 388R primer (GCTGCCTCCCGTAGGAGT) with the Illumina adapter and unique barcode along with Quanta AccuStart II Tough Mix (Quanta bio, Beverly, MA, USA). PCR reactions were conducted on ABI Veriti thermocyclers (Applied Biosystems, Carlsbad, CA, USA) with a thermal profile consisting of 5-min denaturation step at 95 °C, 35 cycles of 94 °C for 30 s, 52 °C for 40 s, and 72 °C for 60 s, and a final extension step of 72 °C for 10 min. PCR products were combined based on qualitative band strength to form the pooled amplicon libraries and size selection was performed using Agencourt AMPure XP beads (Beckman Coulter, Indianapolis, Indiana, USA) and Qiagen Minelute Kits (Qiagen). Pooled libraries were quantified using a Qubit 3.0 fluorometer (Thermo Fisher Scientific, Waltham, MA, USA). Paired read sequencing (2 × 250) was conducted on an Illumina MiSeq (Illumina, San Diego, CA, USA) and using a Reagent Kit v2 Nano, targeting an average ~ 2 k classified reads per sample. Positive controls, negative extraction controls, and no template PCR controls were also included for sequencing.

Bioinformatic curation, quality control filtering, and analysis of data was subsequently performed by RTL Genomics, a division of MicroGenDX. Denoising of sequence reads, chimera detection, and paired read assembly were conducted using Usearch7^9^, UCHIME^10^, and PEAR^11^, respectively. Quality filtered and assembled reads were clustered into operational taxonomic units (OTUs) at 97% sequence similarity threshold using the UPARSE algorithm^12^. OTU assignment then performed using the MicroGenDX reference database. Where possible, taxonomic assignments are reported to the species level as used previously^7^. Multiple sequence alignment and phylogenetic tree construction for downstream analysis performed using MUSCLE^12^, and FastTree^13^. Prior to statistical analysis, additional quality control filtering performed in R to remove OTUs which failed to map to bacterial 16S database and OTUs suspected of being contaminants based on inspection of sequencing controls (i.e., Ralstonia, Pelomonas, and Marinobacter). Total abundance filtering was also used to remove any OTUs found at less than 0.001% of total read counts, which may reflect low level contamination or sequencing artifacts^14^. This methodology has previously been described^8^.

### Statistical analysis

For statistical analysis of baseline participant characteristics, categorical variables are reported as counts and percentages, and continuous variables as means and standard deviations. Chi-squared and Fisher exact tests were used to compare categorical variables, and Student’s t-tests were used for continuous variables. Missing data is reported, where applicable; no imputation techniques or sensitivity analyses were performed.

Coverage of bacterial communities was estimated using Good’s coverage formula^15^. Bacterial alpha diversity was summarized using three indices (OTU richness, Hill1 diversity, and phylogenetic hill1 diversity)^16^. Beta diversity was summarized by Weighted UniFrac^17^ and qualitatively clustered using Principal Coordinate Analysis (PCoA). Alpha and beta diversity assist in interpreting higher level changes in microbial community structure. Alpha diversity measures the diversity in a single sample “within-sample,” whereas beta diversity measures the similarity or dissimilarity, or similarity, of two communities “between-sample”^18^. Samples were characterized as normal or abnormal according to sperm analysis results, with ANOVA or PERMANOVA used to compare each grouping for differences in alpha or beta diversity, respectively. Analysis of composition of Microbiota with Bias Correction (ANCOM-BC) was used to screen bacterial taxa for nonrandom distributions according to sperm analysis findings and reduce false positive rate^19^. Only taxa prevalent in more than 20% of samples were considered (zero_cut = 0.80) and less stringent parameters were used to maximize detection of possible discriminatory taxa (no p-value correction)^8^. Canonical correlation analysis (CCA) was used to measure the associations between bacterial relative abundance of species-level clusters more than 10% prevalent and participant metadata (age, SA volume, SA motility, and SA concentration).

### Ethics

This study was approved by the institutional review board of the University of California, Los Angeles, IRB#21–000,714. All methods were performed in accordance with the relevant guidelines and regulations.

## Results

A total of 73 individuals were included in our study. Participants were then stratified into a priori defined groups: Group 1) normal sperm concentration *and* motility on SA versus at least one abnormality in sperm concentration or motility on SA; for brevity, we will refer to this comparison as “sperm result” in the figures (Table 1); Group 2) normal versus abnormal sperm motility (Supp Table 1); and Group 3) normal versus abnormal sperm concentration (Supp Table 2). To further contextualize this data, Supp Table 3 includes baseline participant characteristics stratified by recruitment groups (infertility versus vasectomy). Study participant characteristics are reported in the above-mentioned tables in detail. The average participant age was 37.94 with a standard deviation of 5.62 and the average BMI was 26.73 with a standard deviation of 6.15. Seventy-eight percent of participants were circumcised. There were no significant differences in age, BMI, circumcision status, smoking, or alcohol intake history among any of the three comparison groups. All participants recruited from the infertility evaluation cohort are engaging in regular sexual activity, whereas the sexual activity of those recruited from the vasectomy consultation cohort was not recorded.

**Table 1:** Baseline Participant Characteristics.

Cohort characteristics	Normal semen analysis group (n = 42)	Abnormal semen analysis group (n = 31)	*p*
Age (yr)	37.25 ± 7.5	37.64 ± 6.7	0.704
Body mass index (kg/m2)	26.84 ± 7.1	26.55 ± 4.5	0.862
Missing Data	8	9	
Circumcised	32 (80%)	21 (75%)	0.625
Missing Data	2	3	
Infertility group	17 (40%)	28 (90%)	** < 0.001**
Smoking status			
Current smoker	0 (0%)	1 (5%)	0.373
Ex-smoker	9 (24%)	6 (27%)	
Never smoker	28 (76%)	15 (68%)	
Missing Data	5	9	
Alcohol use			
None	7 (18%)	4 (20%)	0.999
Social	28 (74%)	16 (80%)	
Heavy	3 (8%)	0 (0%)	
Missing Data	4	11	
Semen analysis			
Semen volume (mL)	2.84 ± 1.4	2.14 ± 1.1	0.024
Semen pH	8.17 ± 0.2	8.12 ± 0.2	0.355
Sperm concentration (million/mL)	80.66 ± 49.0	29.79 ± 49.0	** < 0.001**
% Motile sperm	59.21 ± 11.9	19.35 ± 17.6	** < 0.001**
Total sperm count (million)	201.44 ± 122.7	62.74 ± 94.8	** < 0.001**
Total motile sperm count (million)	116.67 ± 69.0	18.25 ± 30.2	** < 0.001**
% Normal morphology (Kruger strict criteria)	26.17 ± 18.4	2.60 ± 4.7	** < 0.001**
Normozoospermia (> 15 million/mL)	42 (100%)	11 (35%)	** < 0.001**
Oligospermia (< 15 million/mL)	0 (0%)	12 (39%)	
Azoospermia (0 million/mL)	0 (0%)	8 (26%)	

**Figure 1:**
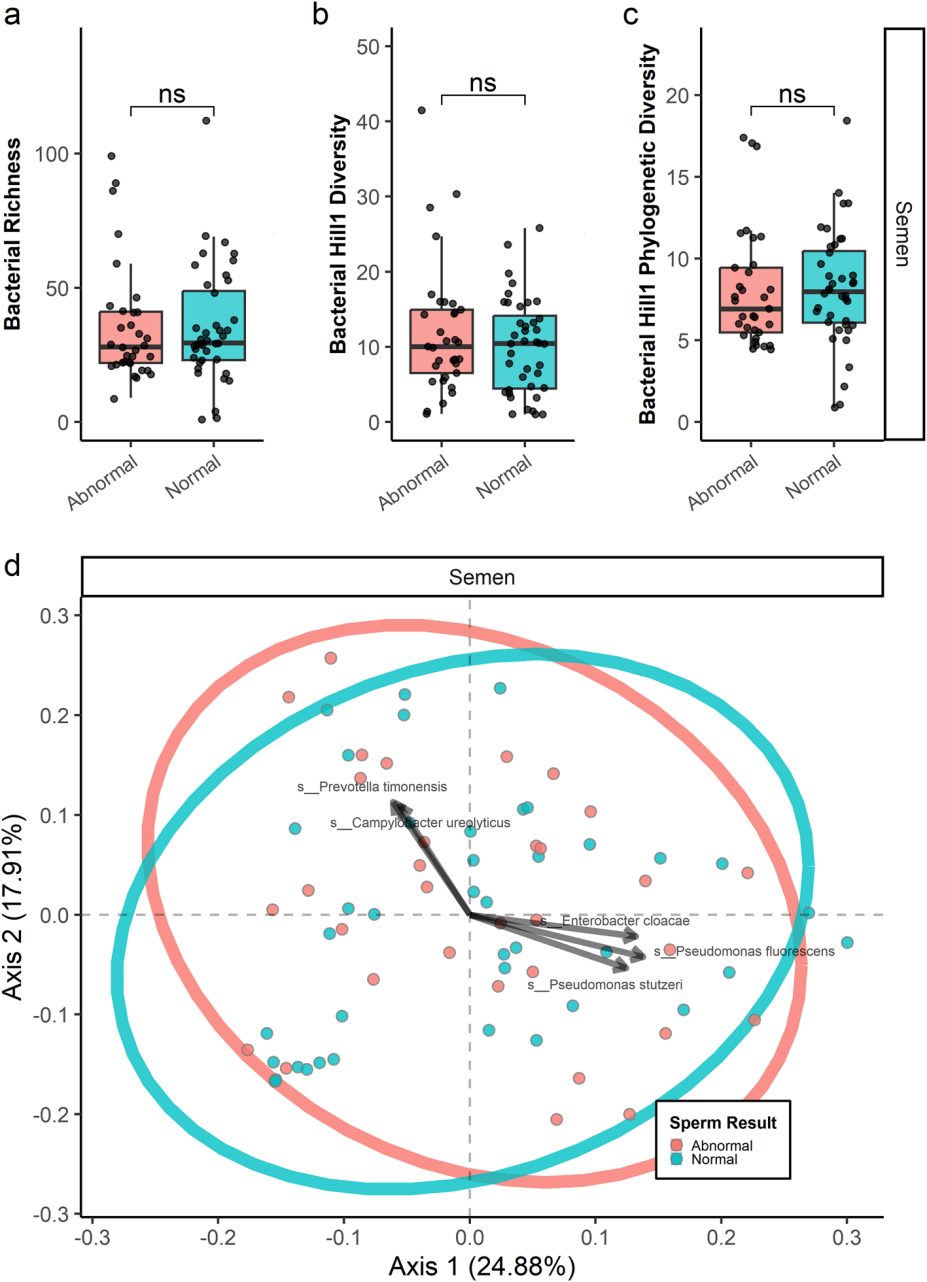
Microbial Community Profiling, Group 1. Global diversity measures comparing participants with normal sperm concentration *and* motility on SA versus participants with at least one abnormality in sperm concentration or motility on SA are outlined in 1A–C (alpha-diversity), and 1D (beta-diversity).

Samples were sequenced to a median depth of 2080 classified reads (Q1 = 1144, Q3 = 5531), providing a median 99.98% Good’s coverage estimate (Q1 = 99.91, Q3 = 100%) and suggesting that samples had been sequenced to sufficient depth (Supp Table 4). There were no significant differences in alpha or beta diversity among any groups investigated in this study. For OTU richness, Hill1 diversity, and phylogenetic hill1 diversity: *p* > 0.10 in all groups examined. This is shown in Fig. 1A–C for the Group 1 comparisons, Supp Figure 1A-C for the Group 2 comparisons, and Supp Figure 2A-C for the Group 3 comparisons. Bacterial communities looked similar when evaluated between group differences using Principal Co-ordinate Analysis with weighted UniFrac distances (Fig. 1D).

**Figure 2:**
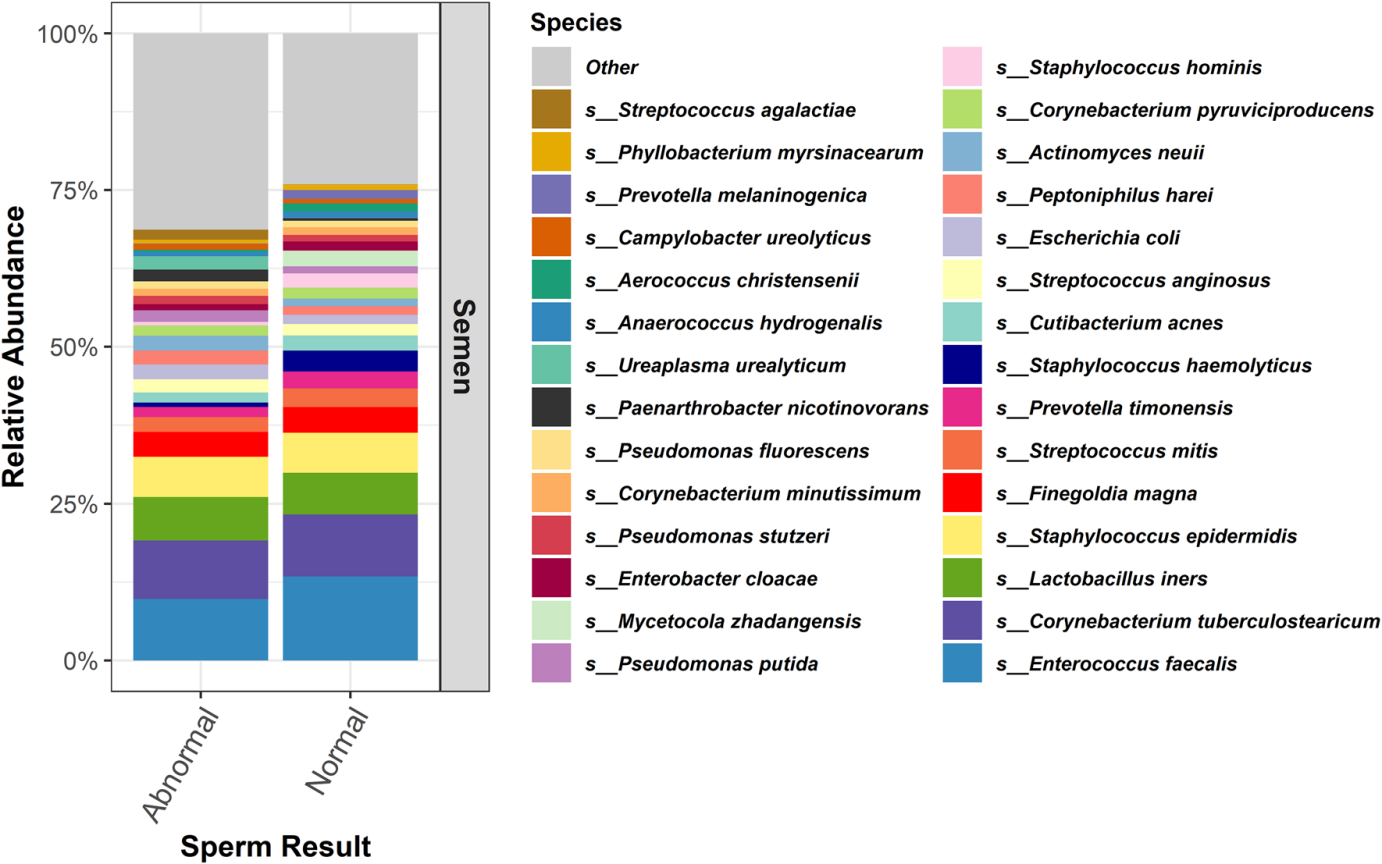
Relative Abundance, Group 1. Relative abundance of the top 30 species of participants with normal sperm concentration *and* motility on SA versus participants with at least one abnormality in sperm concentration or motility on SA.

Regardless of our SA-stratified groupings, there was significant overlap between the most abundant species identified; the top five species always included: *Enterococcus faecalis*, *Corynebacterium tuberculostearicum*, *Lactobacillus iners*, *Staphylococcus epidermidis*, and *Finegoldia magna*. Figure 2, Supp Figure 3, and Supp Figure 4 outline the relative abundance of the top 30 species in the Group 1, 2, and 3 comparisons, respectively.

**Figure 3:**
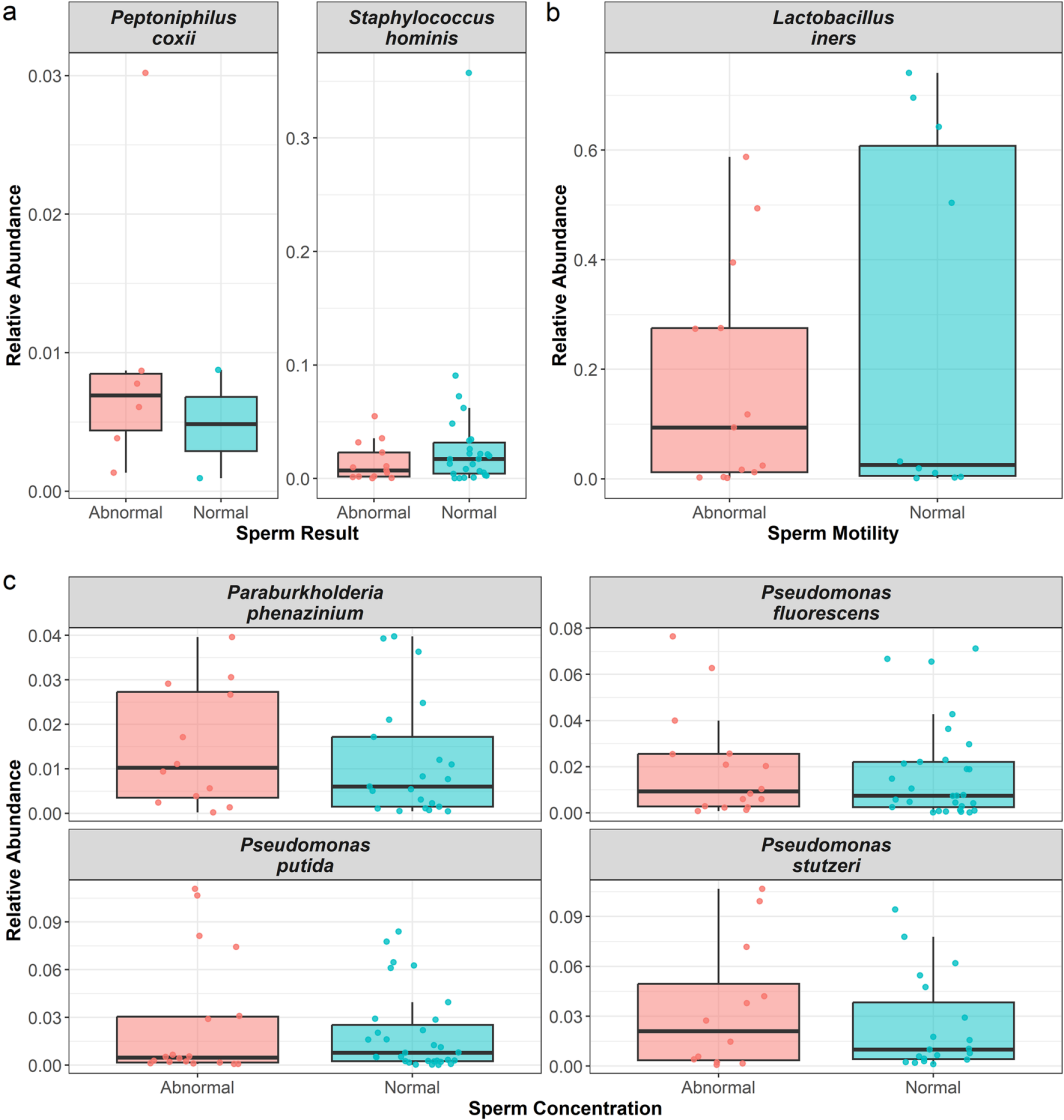
Differential Relative Abundance. Only statistically significant differences are shown. Figure 1A–C, represent the comparisons of groups 1, 2 and 3, respectively.

Using the ANCOM-BC procedure, we found: Group 1) participants with normal SA parameters showed a lower abundance of *Peptoniphilus coxii* (*p* = 0.0469) but a higher abundance of *Staphylococcus hominis* (*p* = 0.00335) compared to participants with any abnormality in sperm concentration or motility; Group 2) participants with normal sperm motility showed a lower abundance of *Lactobacillus iners* (*p* = 0.0464) compared to participants with abnormal sperm motility; Group 3) participants with normal sperm concentration showed a lower abundance of *Paraburkholderia phenazinium* (*p* = 0.0247), *Pseudomonas fluorescens* (*p* = 0.0101), and *Pseudomonas stutzeri* (*p* = 0.0241), but a higher abundance of *Pseudomonas putida* (*p* = 0.00478) compared to participants with anormal sperm concentration (Fig. 3, Supp Table 5).

**Figure 4:**
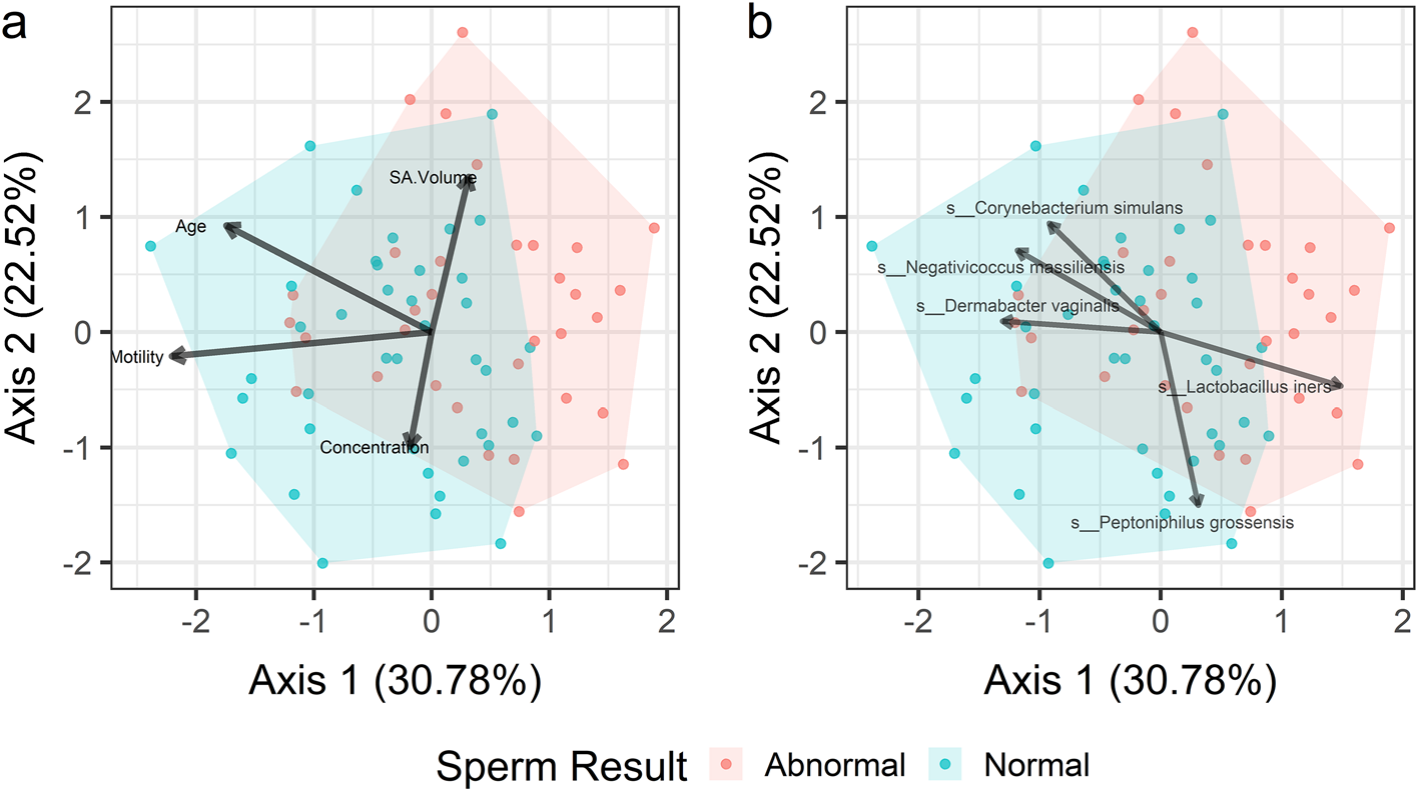
Canonical Correlation Analysis Plot. Visual representation of the canonical correlational analysis, highlighting non-microbiome (3A) and microbiome (3B) variables differentiating participants with normal compared to abnormal SA parameters.

To query beyond our binary analysis of abnormal versus normal SA parameters, we also performed a CCA, which interprets participant data as a continuous variable. All constraining variables (participant age, SA volume, SA concentration, SA motility) were significantly associated with bacterial composition (Supp Table 6). Table 2 outlines the top 20 species with the highest correlation to the two CCA axes that explain the greatest amount of variance in the data. The top five species that account for the greatest degree of variance across the entire data set were: *Lactobacillus iners* (R_cumulative_ = 0.392), *Negativicoccus massiliensis* (R_cumulative_ = 0.379), *Corynebacterium simulans* (R_cumulative_ = 0.374), *Peptinophilus grossensis* (R_cumulative_ = 0.363), and *Dermabacter vaginalis* (R_cumulative_ = 0.280), as visualized in Fig. 4.

**Table 2:** Canonical correlation analysis.

Species	CCA1 P	CCA1 R	CCA2 P	CCA2 R	Cumulative R
*Lactobacillus iners*	0.011	0.297	0.429	− 0.094	0.392
*Negativicoccus massiliensis*	0.044	− 0.237	0.231	0.142	0.379
*Corynebacterium simulans*	0.118	− 0.184	0.109	0.189	0.374
*Peptoniphilus grossensis*	0.602	0.062	0.010	− 0.300	0.363
*Brevibacterium mcbrellneri*	0.134	− 0.177	0.132	0.178	0.355
*Lactobacillus crispatus*	0.231	− 0.142	0.117	0.185	0.327
*Corynebacterium pyruviciproducens*	0.434	− 0.093	0.057	0.224	0.317
*Mobiluncus curtisii*	0.348	0.111	0.091	− 0.199	0.310
*Agrobacterium rhizogenes*	0.468	0.086	0.061	0.220	0.307
*Paraburkholderia phenazinium*	0.231	0.142	0.181	− 0.158	0.300
*Fusobacterium nucleatum*	0.290	0.125	0.159	− 0.167	0.292
*Actinotignum schaalii*	0.146	0.172	0.325	0.117	0.289
*Phyllobacterium myrsinacearum*	0.690	0.047	0.045	0.235	0.283
*Dermabacter vaginalis*	0.025	− 0.262	0.875	0.019	0.280
*Actinomyces radingae*	0.733	− 0.041	0.042	0.239	0.280
*Anaerococcus octavius*	0.135	− 0.176	0.408	0.098	0.275
*Corynebacterium tuberculostearicum*	0.263	− 0.133	0.281	0.128	0.261
*Streptococcus mitis*	0.385	0.103	0.194	− 0.154	0.257
*Helcobacillus massiliensis*	0.312	− 0.120	0.277	0.129	0.249
*Peptoniphilus coxii*	0.083	0.204	0.786	− 0.032	0.237

## Discussion

In this study, we add to the still nascent body of research exploring the relationship between the semen microbiome and fertility. We understand that changes to SA parameters do not necessarily reflect or predict fertility or subfertility, which is an important distinction that should be taken into consideration in the interpretation of this data. Our results suggest that a small group of microorganisms play a critical role in observed alterations of SA parameters. Some of these microbes have been described extensively within other fertility-related contexts, whereas for others, this is the first report where they have been implicated in fertility and subfertility. The present findings do not indicate causality, but these results may inform future mechanistic studies to tease apart the complex relationship between human microbial populations and male fertility.

Aligned with previous studies, our results reveal that semen harbors a diverse, but largely consistent, microbiome. The most abundant members of this niche were *Enterococcus faecalis*, *Staphylococcus epidermidis*, *Corynebacterium tuberculostearicum*, and *Lactobacillus iners*. These findings match with near fidelity to the recently published seminal report by Suarez Arbelaez et al*.*,^8^ which explored taxonomic differences in the semen microbiome in men pre- and post-vasectomy, albeit in a somewhat smaller sample (58 men). Remarkably, we did not identify any significant differences in alpha or beta diversity regardless of our SA-based classifications, suggesting that it may not be a global dysbiosis that contributes to subfertility, but perhaps a more subtle change in certain cornerstone species. Somewhat in contrast to our findings, Lundy and colleagues^5^ recently published a high quality study, that did find differences in alpha and beta diversity in fertile (n = 12) versus infertile men (n = 25). This discordance may reflect different patient populations in addition to methodological differences in analyses.

When taking into consideration semen volume, concentration, and motility, *Lactobacillus iners* emerged as the strongest differentiator between men with normal versus abnormal SA parameters. This finding was then recapitulated in our subgroup analysis, which showed that men with abnormal motility have a semen microbiome particularly enriched in *Lactobacillus iners*. Previously published investigations implicate this species as playing a prominent negative role in fertility; however, much of this literature is related to the vaginal microbiome and female factors. In a study that included 25 couples undergoing the use of assisted reproductive technologies, researchers found that increased abundance of vaginal lavage *Lactobacillus iners* was associated with infertility.^20^ Looking beyond fertility, the relationship between the vaginal microbiome and *Lactobacillus iners* is highly nuanced; in some contexts, this microbe takes on the role of a vaginal symbiont, whereas in others it may worsen vaginal dysbiosis and predispose the host to developing bacterial vaginosis, sexually transmitted infections, and adverse pregnancy outcomes.^21–23^ Our study represents the first report of a negative association between *Lactobacillus iners* and male-factor fertility.

*Lactobacillus iners* has the smallest reported genome of any member of this genus^24^ while also having a subset of enzymes not available to other members of this genus.^25^ For example, *Lactobacillus iners* can produce the less common L-lactic acid (rather than D-lactic acid) isomer, which can induce a pro-inflammatory local milieu. This may be sufficient to impair sperm motility in a clinically meaningful way.^26^ The female literature has even shown associations between the presence of *Lactobacillus iners* and pro-inflammatory cytokines including tumor necrosis factor-alpha and macrophage migration inhibitory factor.^25,27^.

We also found that three species in the genus *Pseudomonas* were differentially abundant when groups were stratified by abnormal and normal sperm concentrations, with *Pseudomonas fluorescens* and *Pseudomonas stutzeri* being more abundant in patients with abnormal SA concentrations, whereas *Pseudomonas putida* less abundant in abnormal SA concentration samples. Previous investigations have reported on the relationship between *Pseudomonas*-predominant genera and low-quality semen.^28^ However, some studies suggest the opposite, with a positive association between genus *Pseudomonas* and total motile sperm counts.^5^ These seemingly conflicting findings in the literature likely reflect, in part, differences in patient populations and sample sizes but also suggest – as do our findings – that not every member of the same genus may function in the same way to impact, be it positively or negatively, fertility.

Adjacent investigations from the female infertility literature found that targeted microbial therapy based off of NGS of the endometrial microbiome improved assisted reproductive technology outcomes in women with recurrent implantation failure.^29^ Though more in its infancy, our findings suggest that there may be similar microbial signatures in semen, which could be worth targeting to improve male factor infertility.

The present study has limitations. Although this work represents the largest semen microbiome sampling in any published work to date, the patient population at our institution is not socioeconomically diverse given the relative affluence of our geographical region. A multi-institutional, longitudinal study design may offer stronger reinforcement or contradiction to our local findings. Collection of semen specimens through masturbation invariably includes inhabitants of the urethral microbiome, which may confound our findings though this still remains the most reasonable method of specimen collection given the alternative of seminal vesicle aspiration is not a practical option. We did not collect information related to recent antibiotic use or genitourinary infections, which may confound our results. Furthermore, we did not collect data on sexual intercourse frequency and whether barrier methods of contraception were used so we are unable to query the impact of potential partner-influenced changes in the microbiome that may have emerged through intercourse. Only a single sample of semen was collected from each participant, which may limit generalizability as the inter-specimen stability of the semen microbiome has not been previously explored. Furthermore, incorporation of additional biomarkers believed to be associated with sperm health such as systemic markers for oxidative stress or DNA fragmentation index may provide important mechanistic context and should be explored in future investigations.

## Conclusions

Our findings highlight a small but critical group of microorganisms that may play an important role in male fertility; namely, *Lactobacillus iners* and members of the genus *Pseudomonas*. This exploratory analysis supports data reported in previously published, smaller, studies while also revealing novel insights that will be critical in guiding future, mechanistic investigations that will help us understand the complex relationship between the semen microbiome and fertility.

### Supplementary Information

Supplementary Tables.

Supplementary Figure 1.

Supplementary Figure 2.

Supplementary Figure 3.

Supplementary Figure 4.

## Data Availability

Raw 16S rRNA sequence data has been uploaded to the SRA under BioProject Accession PRJNA1040881.
