# Supplementary material for: Semen microbiota are dramatically altered in men with abnormal sperm parameters
Source: Sci Rep. 2024 Jan 11;14:1068. doi: 10.1038/s41598-024-51686-4 (PMC10784508; doi:10.1038/s41598-024-51686-4)
Supplement: Supplementary file 1 — Supplementary Tables. [file 41598_2024_51686_MOESM1_ESM.docx]

Supp Table 1. Participant Demographics and Semen Analysis Parameters for Men with Normal and Abnormal Sperm Motility

| **Cohort Characteristics** | **Normal Motility Group (n = 46)** | **Abnormal Motility Group (n = 27)** | ***p*** |
| --- | --- | --- | --- |
| **Age (yr)** | 37.88 ± 4.7 | 38.04 ± 6.9 | 0.908 |
| **Body mass index (kg/m2)** | 26.76 ± 6.9 | 26.66 ± 4.7 | 0.955 |
| **Missing Data** | 10 | 7 |  |
| **Circumcised** | 36 (82%) | 17 (71%) | 0.296 |
| **Missing Data** | 2 | 3 |  |
| **Infertility group** | 21 (46%) | 24 (89%) | **< 0.001** |
| **Smoking status** |  |  |  |
| **Current smoker** | 0 (0%) | 1 (6%) | 0.317 |
| **Ex-smoker** | 11 (27%) | 4 (22%) |  |
| **Never smoker** | 30 (73%) | 13 (72%) |  |
| **Missing Data** | 5 | 9 |  |
| **Alcohol use** |  |  |  |
| **None** | 7 (18%) | 4 (22%) | 0.724 |
| **Social** | 30 (75%) | 14 (78%) |  |
| **Heavy** | 3 (8%) | 0 (0%) |  |
| **Missing Data** | 6 | 9 |  |
| **Semen analysis** |  |  |  |
| **Semen volume (mL)** | 2.86 ± 1.4 | 2.01 ± 1.1 | 0.007 |
| **Semen pH** | 8.17 ± 0.2 | 8.12 ± 0.2 | 0.863 |
| **Sperm concentration (million/mL)** | 74.06 ± 51.6 | 33.51 ± 51.5 | **0.002** |
| **% Motile sperm** | 58.43 ± 11.7 | 14.78 ± 13.7 | **< 0.001** |
| **Total sperm count (million)** | 185.26 ± 128.6 | 69.76 ± 99.7 | **< 0.001** |
| **Total motile sperm count (million)** | 107.17 ± 72.8 | 19.83 ± 5.6 | **< 0.001** |
| **% Normal morphology (Kruger strict criteria)** | 24.99 ± 18.8 | 5.35 ± 5.6 | **< 0.001** |
| **Normozoospermia (>15 million/mL)** | 42 (91%) | 11 (41%) | **< 0.001** |
| **Oligospermia (<15 million/mL)** | 4 (9%) | 8 (30%) |  |
| **Azoospermia (0 million/mL)** | 0 (0%) | 8 (30%) |  |

Supp Table 2. Participant Demographics and Semen Analysis Parameters for Men with Normal and Abnormal Sperm Concentration

| **Cohort Characteristics** | **Normal Concentration Group (n = 53)** | **Abnormal Concentration Group (n = 20)** | ***p*** |
| --- | --- | --- | --- |
| **Age (yr)** | 38.46 ± 5.5 | 36.57 ± 5.9 | 0.102 |
| **Body mass index (kg/m2)** | 26.48 ± 6.6 | 27.41 ± 5.0 | 0.310 |
| **Missing Data** | 12 | 5 |  |
| **Circumcised** | 39 (78%) | 14 (78%) | 0.984 |
| **Missing Data** | 3 | 2 |  |
| **Infertility Group** | 25 (47%) | 20 (100%) | **< 0.001** |
| **Smoking status** |  |  |  |
| **Current smoker** | 0 (0%) | 1 (9%) | 0.187 |
| **Ex-smoker** | 13 (27%) | 2 (18%) |  |
| **Never smoker** | 35 (73%) | 8 (73%) |  |
| **Missing Data** | 5 | 9 |  |
| **Alcohol use** |  |  |  |
| **None** | 8 (17%) | 3 (27%) | 0.421 |
| **Social** | 36 (77%) | 8 (73%) |  |
| **Heavy** | 3 (6%) | 0 (0%) |  |
| **Missing Data** | 6 | 9 |  |
| **Semen analysis** |  |  |  |
| **Semen volume (mL)** | 2.70 ± 1.3 | 2.13 ± 1.2 | 0.096 |
| **Semen pH** | 8.15 ± 0.2 | 8.13 ± 0.2 | 0.738 |
| **Sperm concentration (million/mL)** | 80.36 ± 50.0 | 2.61 ± 3.6 | **< 0.001** |
| **% Motile sperm** | 51.96 ± 18.3 | 16.65 ± 20.4 | **< 0.001** |
| **Total sperm count (million)** | 193.80 ± 117.6 | 6.70 ± 10.7 | **< 0.001** |
| **Total motile sperm count (million)** | 102.22 ± 69.3 | 2.40 ± 4.4 | **< 0.001** |
| **% Normal morphology (Kruger strict criteria)** | 22.23 ± 18.2 | 0.27 ± 0.2 | **< 0.001** |
| **Normozoospermia (>15 million/mL)** | 53 (100%) | 0 (0%) | **< 0.001** |
| **Oligospermia (<15 million/mL)** | 0 (0%) | 12 (60%) |  |
| **Azoospermia (0 million/mL)** | 0 (0%) | 8 (40%) |  |

Supp Table 3. Participant Demographics and Semen Analysis Parameters Based on Recruitment Group

| **Cohort Characteristics** | **Vasectomy Group (n = 28)** | **Infertility Group (n = 45)** | ***p*** |
| --- | --- | --- | --- |
| **Age (yr)** | 40.30 ± 5.5 | 36.52 ± 5.3 | **0.005** |
| **Body mass index (kg/m2)** | 27.05 ± 8.4 | 26.52 ± 4.2 | 0.757 |
| **Missing Data** | 6 | 11 |  |
| **Circumcised** | 23 (82%) | 30 (75%) | 0.484 |
| **Missing Data** | 0 | 5 |  |
| **Infertility group** | 0 (0%) | 45 (100%) | **< 0.001** |
| **Smoking status** |  |  |  |
| **Current smoker** | 0 (0%) | 1 (3%) | 0.999 |
| **Ex-smoker** | 7 (28%) | 8 (24%) |  |
| **Never smoker** | 18 (72%) | 25 (74%) |  |
| **Missing Data** | 3 | 11 |  |
| **Alcohol use** |  |  |  |
| **None** | 3 (13%) | 8 (24%) | 0.333 |
| **Social** | 19 (79%) | 25 (74%) |  |
| **Heavy** | 2 (8%) | 1 (3%) |  |
| **Missing Data** | 4 | 11 |  |
| **Semen analysis** |  |  |  |
| **Semen volume (mL)** | 2.48 ± 1.2 | 2.59 ± 1.4 | 0.749 |
| **Semen pH** | 8.17 ± 0.3 | 8.13 ± 0.2 | 0.489 |
| **Sperm concentration (million/mL)** | 80.29 ± 44.7 | 45.85 ± 56.9 | **0.008** |
| **% Motile sperm** | 57.36 ± 17.0 | 32.91 ± 24.0 | **< 0.001** |
| **Total sperm count (million)** | 189.40 ± 124.6 | 113.38 ± 127.1 | **0.015** |
| **Total motile sperm count (million)** | 109.97 ± 73.7 | 53.04 ± 66.13 | **0.001** |
| **% Normal morphology (Kruger strict criteria)** | 25.65 ± 20.63 | 10.26 ± 14.2 | **< 0.001** |
| **Normozoospermia (>15 million/mL)** | 28 (100%) | 25 (56%) | **< 0.001** |
| **Oligospermia (<15 million/mL)** | 0 (0%) | 12 (27%) |  |
| **Azoospermia (0 million/mL)** | 0 (0%) | 8 (18%) |  |

Supp Table 4. Sequence Depth

| Sample | Singletons | Reads | Goods Coverage |
| --- | --- | --- | --- |
| 547124 | 1 | 703 | 99.85775249 |
| 547125 | 1 | 8240 | 99.98786408 |
| 547126 | 3 | 16720 | 99.98205742 |
| 547258 | 1 | 1156 | 99.91349481 |
| 547259 | 0 | 954 | 100 |
| 547260 | 3 | 1447 | 99.7926745 |
| 547261 | 0 | 778 | 100 |
| 552328 | 1 | 2152 | 99.9535316 |
| 552329 | 1 | 13659 | 99.99267882 |
| 552330 | 2 | 10615 | 99.98115874 |
| 552331 | 2 | 9438 | 99.97880907 |
| 552332 | 2 | 5816 | 99.9656121 |
| 552333 | 2 | 2503 | 99.92009588 |
| 552334 | 1 | 10002 | 99.990002 |
| 553429 | 0 | 1916 | 100 |
| 553432 | 1 | 912 | 99.89035088 |
| 553434 | 2 | 1830 | 99.89071038 |
| 553438 | 2 | 1965 | 99.89821883 |
| 555261 | 8 | 10467 | 99.92356931 |
| 555262 | 0 | 2791 | 100 |
| 555263 | 3 | 2535 | 99.8816568 |
| 555264 | 0 | 2834 | 100 |
| 555702 | 1 | 683 | 99.85358712 |
| 555704 | 0 | 1087 | 100 |
| 555706 | 0 | 1084 | 100 |
| 555708 | 3 | 897 | 99.66555184 |
| 556960 | 1 | 18001 | 99.99444475 |
| 556966 | 0 | 1286 | 100 |
| 556969 | 0 | 1632 | 100 |
| 556977 | 1 | 4483 | 99.97769351 |
| 556978 | 2 | 799 | 99.74968711 |
| 557423 | 1 | 744 | 99.8655914 |
| 557425 | 0 | 2199 | 100 |
| 557428 | 2 | 1243 | 99.83909895 |
| 568417 | 0 | 1131 | 100 |
| 568419 | 0 | 628 | 100 |
| 568426 | 1 | 1537 | 99.93493819 |
| 568427 | 0 | 960 | 100 |
| 569535 | 6 | 7065 | 99.91507431 |
| 569537 | 1 | 1793 | 99.94422755 |
| 569538 | 1 | 3998 | 99.97498749 |
| 570672 | 1 | 1665 | 99.93993994 |
| 570674 | 0 | 3747 | 100 |
| 573138 | 0 | 4441 | 100 |
| 573139 | 1 | 6664 | 99.984994 |
| 573141 | 3 | 3435 | 99.91266376 |
| 574773 | 2 | 2484 | 99.9194847 |
| 574775 | 1 | 1552 | 99.93556701 |
| 574778 | 0 | 2080 | 100 |
| 576240 | 0 | 5526 | 100 |
| 576242 | 0 | 5620 | 100 |
| 576244 | 1 | 1428 | 99.92997199 |
| 576246 | 0 | 654 | 100 |
| 577276 | 0 | 3709 | 100 |
| 577278 | 8 | 2258 | 99.64570416 |
| 577280 | 0 | 12080 | 100 |
| 577283 | 1 | 5535 | 99.98193315 |
| 578789 | 1 | 525 | 99.80952381 |
| 578791 | 2 | 16665 | 99.9879988 |
| 581426 | 1 | 6264 | 99.98403576 |
| 581427 | 3 | 1269 | 99.76359338 |
| 586590 | 0 | 1755 | 100 |
| 586591 | 2 | 5869 | 99.96592264 |
| 591546 | 4 | 3333 | 99.879988 |
| 591548 | 0 | 1216 | 100 |
| 591562 | 3 | 1056 | 99.71590909 |
| 591563 | 0 | 1082 | 100 |
| 607986 | 0 | 1099 | 100 |
| 607989 | 1 | 2671 | 99.96256084 |
| 611922 | 0 | 1427 | 100 |
| 611924 | 1 | 11834 | 99.99154977 |
| 616777 | 2 | 1338 | 99.85052317 |
| 616778 | 6 | 3556 | 99.83127109 |

Supp Table 5. Differential Abundance of Microorganisms by Group

| **Group 1; Normal Versus Abnormal SA Parameters** | | | | | | |
| --- | --- | --- | --- | --- | --- | --- |
| **Species** | **beta** | **p** | **SE** | **W** | **Normal** | **Abnormal** |
| *Staphylococcus hominis* | 1.201 | 0.003 | 0.409 | 2.934 | 0.017 | 0.007 |
| *Peptoniphilus coxii* | -0.484 | 0.047 | 0.244 | -1.987 | 0.005 | 0.007 |
|  |  |  |  |  |  |  |
| **Group 2; Normal Versus Abnormal Sperm Motility** | | | | | | |
| **Species** | **beta** | **p** | **SE** | **W** | **Normal** | **Abnormal** |
| *Lactobacillus iners* | -1.313 | 0.046 | 0.659 | -1.992 | 0.026 | 0.094 |
|  |  |  |  |  |  |  |
| **Group 3; Normal Versus Abnormal Sperm Concentration** | | | | | | |
| **Species** | **beta** | **p** | **SE** | **W** | **Normal** | **Abnormal** |
| *Pseudomonas fluorescens* | -1.092 | 0.010 | 0.424 | -2.573 | 0.007 | 0.009 |
| *Pseudomonas putida* | -1.014 | 0.020 | 0.435 | -2.330 | 0.008 | 0.005 |
| *Pseudomonas stutzeri* | -1.069 | 0.024 | 0.474 | -2.255 | 0.010 | 0.021 |
| *Paraburkholderia phenazinium* | -0.887 | 0.025 | 0.395 | -2.246 | 0.006 | 0.010 |

Supp Table 6. Constraining Variables for Canonical Correlation Analysis

| **Constraining Variable** | **R** | **P** |
| --- | --- | --- |
| **Age** | 0.395 | 0.001 |
| **Semen Analysis** |  |  |
| **Motility** | 0.443 | 0.001 |
| **Concentration** | 0.202 | 0.042 |
| **Volume** | 0.277 | 0.013 |
