# Supplementary figures and images for: Semen microbiota are dramatically altered in men with abnormal sperm parameters
Source: Sci Rep. 2024 Jan 11;14:1068. doi: 10.1038/s41598-024-51686-4 (PMC10784508; doi:10.1038/s41598-024-51686-4)

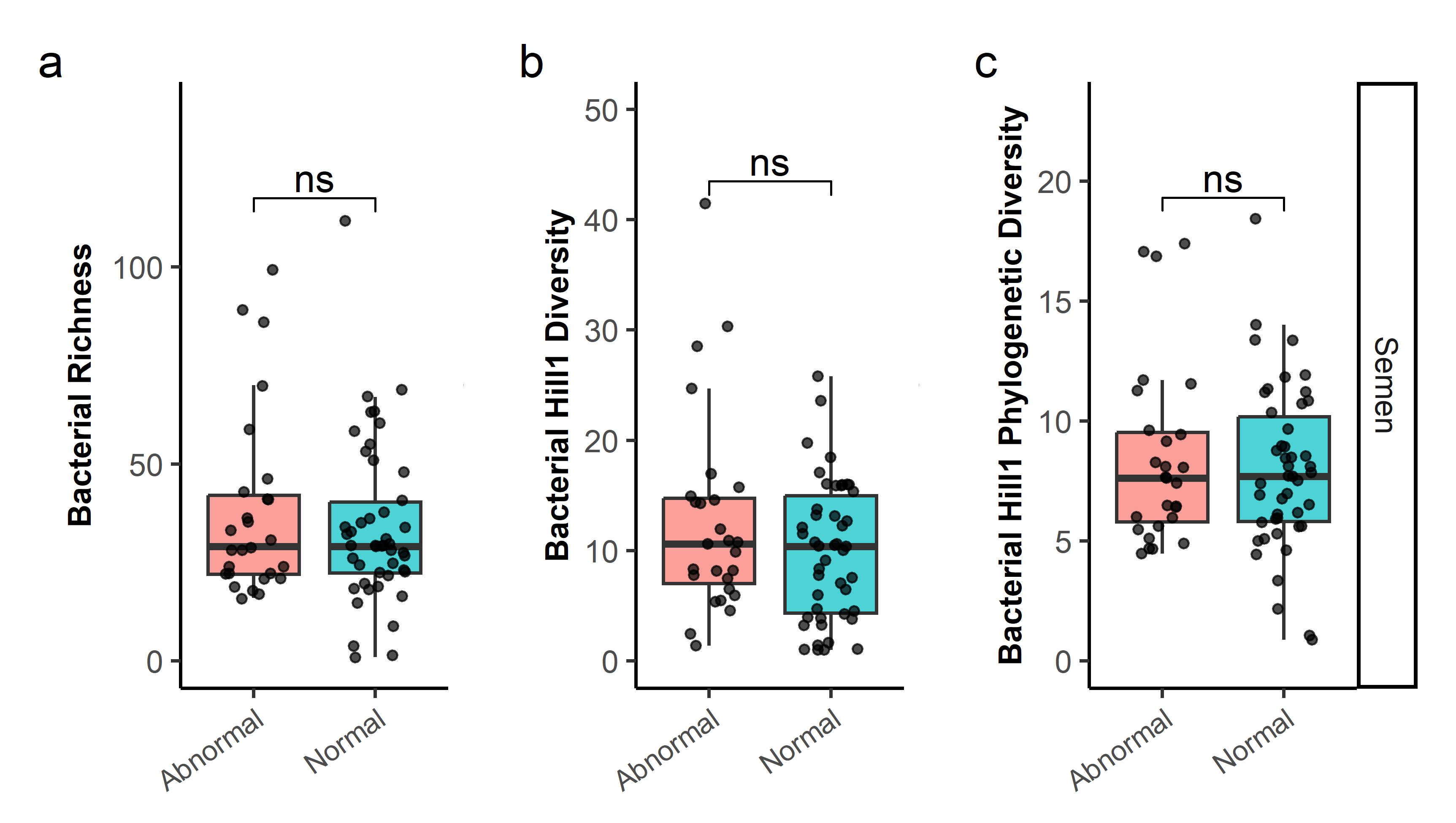

Supplement: Supplementary file 2 — Supplementary Figure 1. [file 41598_2024_51686_MOESM2_ESM.png]

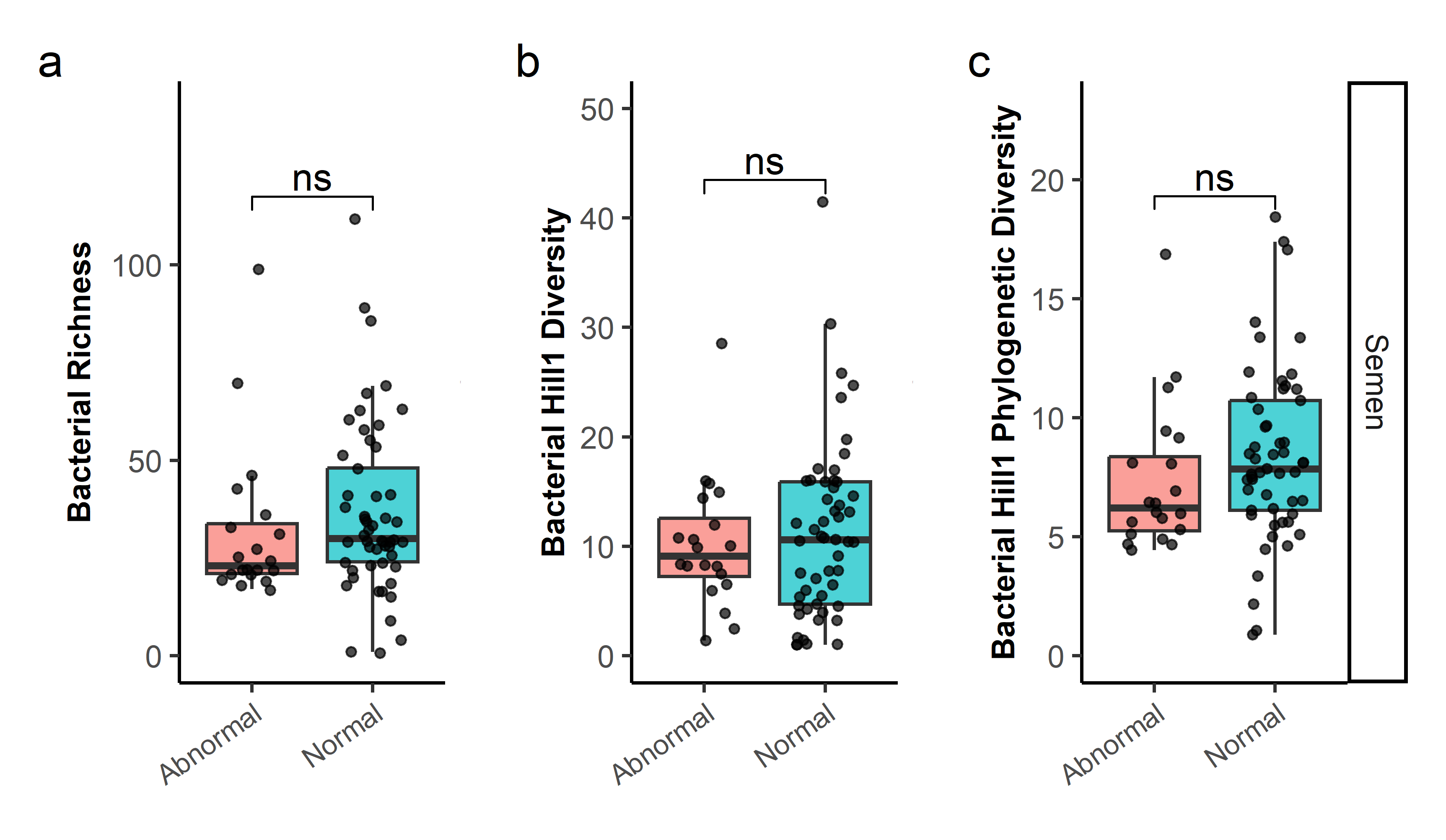

Supplement: Supplementary file 3 — Supplementary Figure 2. [file 41598_2024_51686_MOESM3_ESM.png]

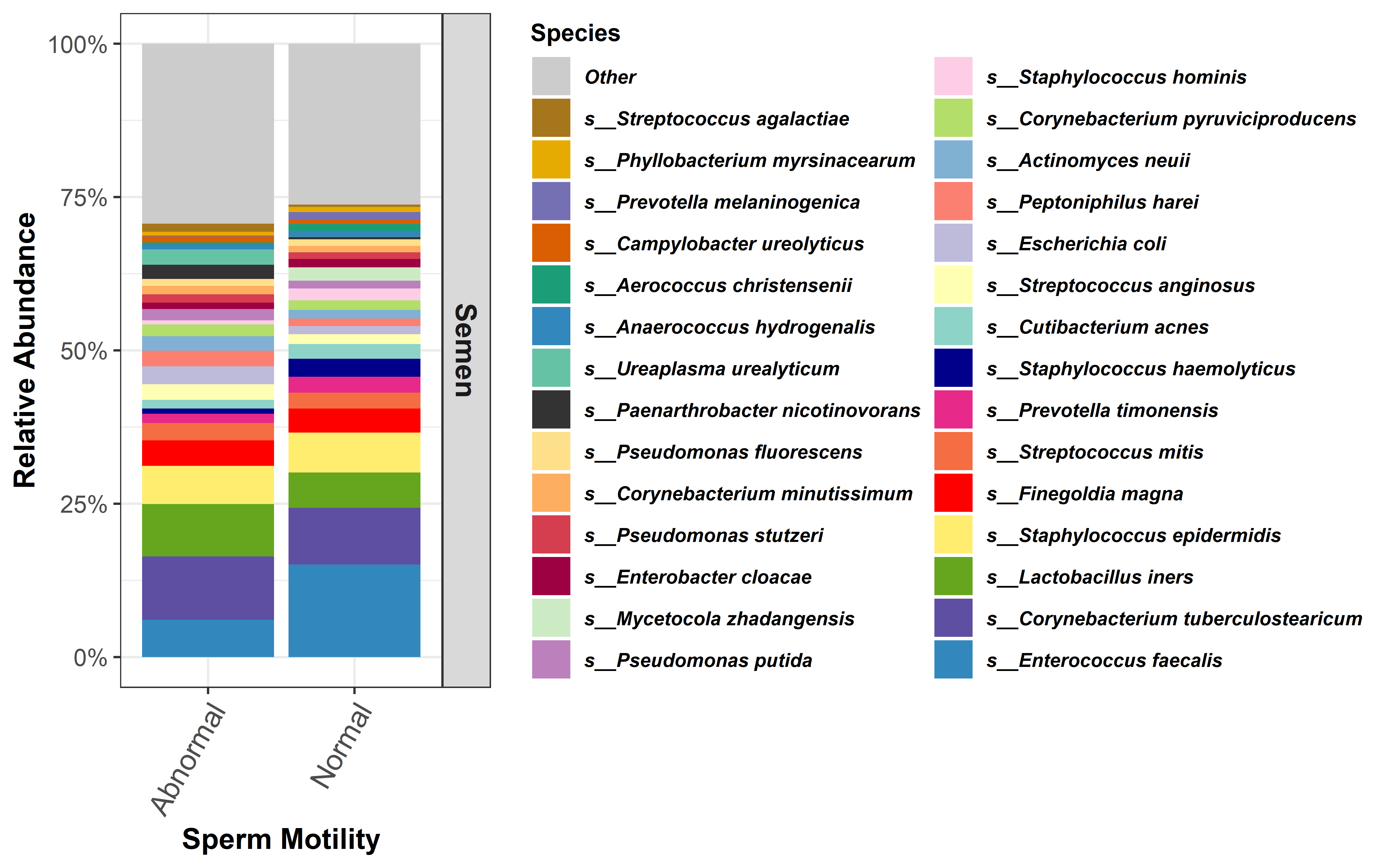

Supplement: Supplementary file 4 — Supplementary Figure 3. [file 41598_2024_51686_MOESM4_ESM.tiff]

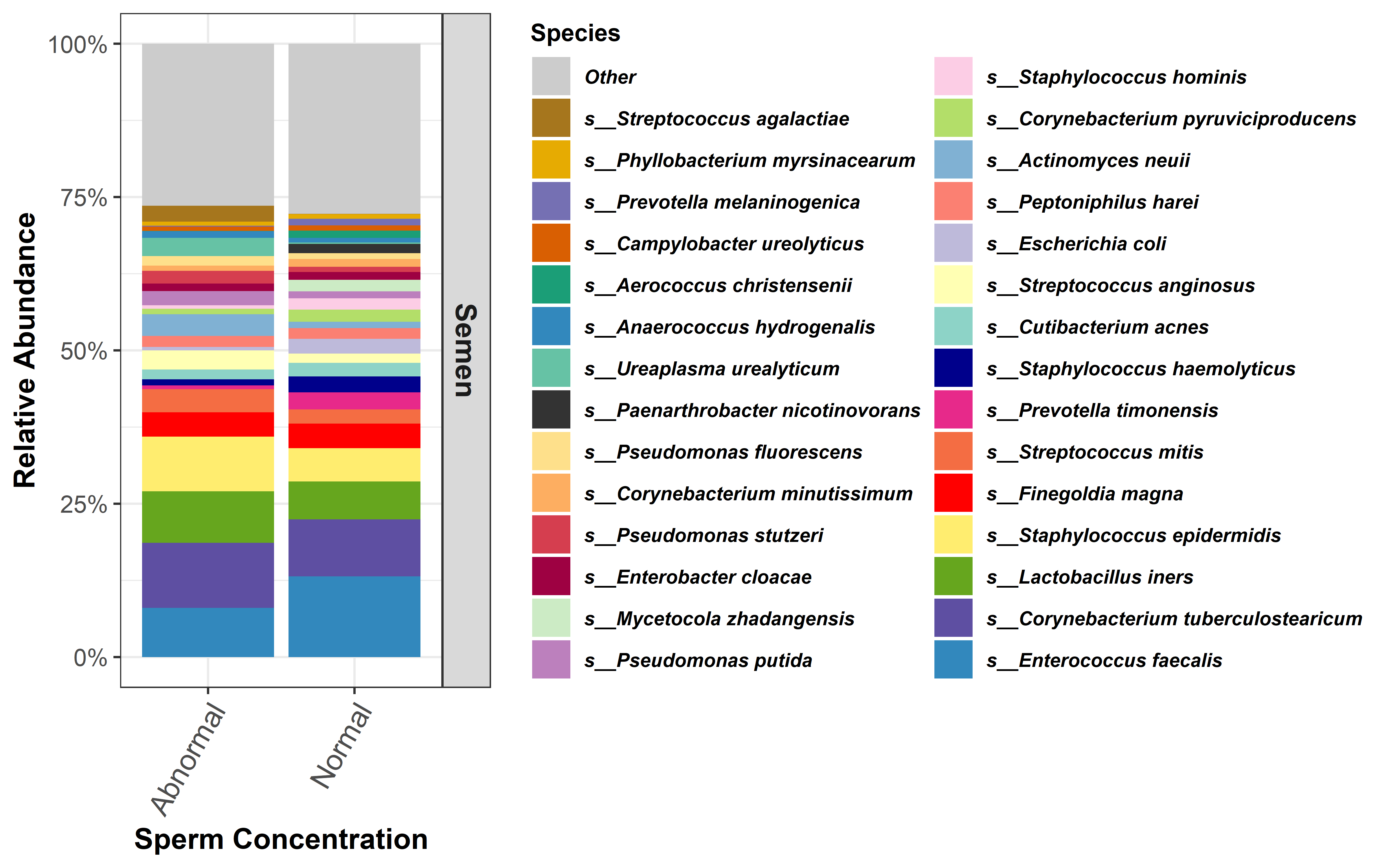

Supplement: Supplementary file 5 — Supplementary Figure 4. [file 41598_2024_51686_MOESM5_ESM.tiff]
